# Monoclonal Gammopathy in Autoimmune Diseases: Marker, Consequence, Pathogenetic Factor—Or Coincidence?

**DOI:** 10.3390/biomedicines14102288

**Published:** 2026-10-09

**Authors:** Györgyi Műzes, Ferenc Sipos

**Affiliations:** Immunology Division, Department of Internal Medicine and Hematology, Semmelweis University, 1088 Budapest, Hungary

**Keywords:** monoclonal gammopathy, MGUS, autoimmune diseases, Sjögren’s syndrome, rheumatoid arthritis, systemic lupus erythematosus, multiple myeloma, B cell activation

## Abstract

The possible association between monoclonal gammopathy and autoimmune/immune-mediated diseases has received increasing attention in recent years. Several observational studies have reported an increased prevalence of monoclonal gammopathy in certain autoimmune conditions, particularly primary Sjögren’s syndrome, rheumatoid arthritis, and systemic lupus erythematosus. However, recent population-based screening data from the iStopMM study found no significant association between autoimmune disease and screen-detected MGUS after adjustment for age and sex, suggesting that ascertainment bias may partly explain associations observed in clinically identified cohorts. The underlying mechanisms may include chronic antigen stimulation, sustained B-cell activation, germinal-center dysregulation, increased T follicular helper cell activity, and BAFF/APRIL-, IL-6-, NF-κB-, and STAT3-mediated signaling pathways. Based on current evidence, the relationship between monoclonal gammopathy and autoimmunity can be interpreted according to four potentially overlapping models: monoclonal gammopathy may serve as a marker of chronic immune activation, develop as a consequence of persistent autoimmune inflammation, act as a pathogenetic factor through the direct effects of the monoclonal immunoglobulin or the underlying clonal B-cell/plasma-cell population, or represent a coincidental finding related to factors such as age, genetic background, or increased diagnostic surveillance. This review summarizes the major epidemiological, pathogenetic, and clinical evidence supporting these interpretations and discusses their potential diagnostic, prognostic, and therapeutic implications. Finally, we highlight key directions for future research, including longitudinal single-cell and spatial omics approaches, further characterization of the BAFF/APRIL axis, and investigation of PANoptosis as a potential link between chronic inflammation and clonal plasma-cell disorders.

## 1. Introduction

In monoclonal gammopathy, a single clonal plasma cell population produces immunoglobulin or immunoglobulin fragments of identical structure, known as M-proteins (paraproteins). The spectrum of this condition ranges from completely asymptomatic, premalignant states to overt hematologic malignancies. The most common form is monoclonal gammopathy of undetermined significance (MGUS), which is considered a precursor to multiple myeloma (MM) and other lymphoid malignancies [1,2].

Over time, some cases of MGUS may progress to smoldering myeloma (SMM) and then to clinically manifest MM. In cases where monoclonal immunoglobulin causes organ damage or clinical symptoms without meeting the diagnostic criteria for multiple myeloma, the term “monoclonal gammopathy of clinical significance” (MGCS) is used [3,4,5].

MGUS is the most common premalignant plasma-cell disorder. In the population-based study by Kyle et al., MGUS was identified in 3.2% of individuals aged ≥50 years, and its prevalence increased progressively with age, reaching 5.3% among individuals aged ≥70 years and 7.5% among those aged ≥85 years [1]. Smoldering multiple myeloma (SMM) is less common than MGUS; in the population-based iStopMM screening study, its prevalence was 0.53% (95% CI, 0.49–0.57%) among individuals aged ≥40 years [6]. However, its risk of progression to symptomatic MM is substantially higher than that of MGUS [7]. The incidence of multiple myeloma is on the rise worldwide, partly as a result of population aging [3].

Research has recently confirmed that the progression from MGUS to SMM to MM is not merely a series of genetic events but rather a complex process based on interactions between clonal plasma cells, the immune system, and the bone marrow microenvironment. This insight has significantly contributed to the development of modern risk assessment models and the exploration of opportunities for early therapeutic intervention [3,7].

In recent years, the relationship between monoclonal gammopathies and autoimmune/immune-mediated diseases has attracted increasing scientific interest. The primary focus of this review is the occurrence and significance of monoclonal gammopathy in patients with pre-existing autoimmune or immune-mediated conditions, rather than a comprehensive review of autoimmune manifestations caused by monoclonal gammopathies. Nevertheless, disorders in which the monoclonal immunoglobulin or the underlying B-cell/plasma-cell clone directly contributes to immune-mediated manifestations are discussed selectively because they represent the ‘pathogenetic factor’ component of our proposed conceptual framework. In this context, the recently introduced concept of monoclonal autoimmune gammopathies (MAGa) provides a particularly relevant example [8]. Numerous epidemiological studies have confirmed that the prevalence of paraproteinemia and monoclonal gammopathy in certain autoimmune diseases exceeds the frequency observed in the general population [9].

In particular, increased clonal B cell activation, paraprotein production, and a concomitant elevated risk of hematologic malignancies have been observed in cases of primary Sjögren’s syndrome (SS), rheumatoid arthritis (RA), and systemic lupus erythematosus (SLE) [10,11]. In primary SS, the presence of monoclonal gammopathy is associated with increased disease activity as well as an elevated risk of lymphoma and myeloma [10]. Several mechanisms may underlie this association. Prolonged autoantigen exposure and chronic immune activation may result in continuous B cell stimulation, which promotes clonal plasma cell expansion. On the other hand, immunosuppressive and biologic therapies used in the treatment of autoimmune diseases may also influence the immune system’s tumor-suppressing mechanisms [9].

Among the newer concepts is “monoclonal gammopathy of rheumatologic significance” (MGRhS): this term refers to clonal B cell abnormalities that do not meet the diagnostic criteria for a hematologic malignancy but are capable of causing multiorgan damage or influencing the management of rheumatologic conditions [12].

Based on current knowledge, it remains unclear whether monoclonal gammopathy is a biomarker of autoimmune processes, a consequence of them, a pathogenic factor, or an independent phenomenon determined by shared risk factors. According to some observations, the appearance of M-protein may be a marker of chronic antigen stimulation and immune activation, which is associated with disease activity and an increased risk of developing hematologic malignancies later on [10]. Other studies suggest that the inflammatory microenvironment may directly contribute to the formation and persistence of clonal plasma cell populations [13]. Furthermore, it cannot be ruled out that some of the observed associations are explained by common risk factors—such as age, genetic background, or increased diagnostic activity. Accordingly, the relationship between monoclonal gammopathies and autoimmune diseases is likely heterogeneous, and different pathomechanisms may underlie the individual clinical conditions [9]. Importantly, recent evidence from the population-based iStopMM screening study challenges the assumption of a uniform association between autoimmune disease and MGUS: among 75,422 systematically screened individuals, autoimmune disease was not significantly associated with screen-detected MGUS after adjustment for age and sex (PR 1.05, 95% CI 0.97–1.15), highlighting the potential influence of ascertainment bias in earlier clinically identified cohorts [14].

The central question of this article is therefore not merely whether monoclonal gammopathy is more common in autoimmune diseases but rather what this monoclonality signifies in a given patient. Our aim is the review, based on available epidemiological, pathophysiological, and clinical knowledge, of whether monoclonal gammopathy can be considered a marker, a consequence, or a pathogenic factor of autoimmune diseases, or whether it is only a coincidence.

### Literature Search Strategy

This narrative review was based on a literature search of PubMed/MEDLINE, Scopus, and Web of Science for articles published from database inception to August 2026. Search terms included combinations of ‘monoclonal gammopathy’, ‘MGUS’, ‘monoclonal gammopathy of clinical significance’, ‘multiple myeloma’, ‘autoimmune disease’, ‘immune-mediated disease’, ‘autoinflammatory disease’, ‘rheumatoid arthritis’, ‘systemic lupus erythematosus’, ‘Sjögren’s syndrome’, ‘autoimmune hepatitis’, ‘primary biliary cholangitis’, ‘psoriasis’, ‘IgG4-related disease’, ‘chronic infection’, and related terms. English-language original studies, systematic reviews, meta-analyses, relevant guidelines, and selected mechanistic studies were considered. References of retrieved articles were also screened to identify additional relevant publications. Studies were selected on the basis of their relevance to the epidemiological, pathogenetic, prognostic, and clinical relationship between monoclonal gammopathy and autoimmune/immune-mediated disorders. Studies were included if they provided epidemiological, mechanistic, prognostic, or clinically relevant evidence concerning the relationship between monoclonal gammopathy and autoimmune/immune-mediated disease. Priority was given to population-based studies, cohort and case–control studies, systematic reviews and meta-analyses, consensus or guideline documents, and mechanistic studies directly relevant to the proposed biological models. Case reports and small case series were considered primarily for rare disease associations or emerging clinical entities for which higher-level evidence was unavailable. Studies were excluded if they did not specifically address monoclonal gammopathy or related clonal B-cell/plasma-cell disorders, lacked relevance to autoimmune/immune-mediated disease, or provided insufficient information to evaluate the reported association. Given the narrative nature of this review, no formal meta-analysis or quantitative evidence synthesis was performed.

## 2. Aspects of Pathogenesis

### 2.1. Immune System Homeostasis and B-Cell–Plasma Cell Differentiation

#### 2.1.1. The Effects of Chronic Antigen Stimulation

Immune homeostasis requires a balance between effective immune defense and immunological tolerance. Disruption of this regulated system could lead to the development of autoimmune, lymphoproliferative, or neoplastic conditions [15].

Under physiological conditions, antigen exposure induces sequential extrafollicular and germinal-center B-cell responses. The initial extrafollicular response generates short-lived plasma cells that provide rapid humoral immunity. This is followed by affinity maturation in the germinal centers, resulting in the formation of long-lasting plasma cells and memory B cells. Long-lived plasma cells can persist in the bone marrow for years and contribute to sustained humoral immunity [15,16].

Prolonged antigenic stimulation, however, may disrupt plasma-cell homeostasis. During persistent inflammation and continued antigen exposure, the generation of short-lived plasma cells increases, whereas the maintenance of long-lived plasma cells may be impaired. This imbalance may partly result from cytokine-mediated alterations in the bone marrow microenvironment, particularly those involving TNF-α, which can impair signals required for plasma-cell survival [16]. In autoimmune diseases, sustained immune activation and repeated antigen exposure may promote the emergence and persistence of clonal B-cell populations.

#### 2.1.2. Autoantigens and Polyclonal Activation

Persistent exposure to autoantigens is a characteristic feature of autoimmune diseases and can drive sustained B-cell activation and antibody production. Chronic antigen stimulation initially induces a polyclonal response, but over time, certain clones may gain a proliferative advantage, resulting in oligoclonal and then monoclonal expansion [17]. Supporting an antigen-driven model in at least a subset of cases, the monoclonal immunoglobulin produced by MGUS or myeloma plasma cells may recognize specific microbial or self-antigens, suggesting that persistent antigenic stimulation can provide a selective advantage to particular B-cell clones [18,19]. However, this mechanism is unlikely to explain all cases of monoclonal gammopathy, as studies of paraprotein antigen specificity have yielded heterogeneous results and have not consistently supported a universal causal role for chronic antigenic stimulation [19].

In recent years, increasing attention has been directed toward asymptomatic clonal precursor states across different hematopoietic lineages, including monoclonal B-cell lymphocytosis (MBL), monoclonal gammopathy of undetermined significance (MGUS), and T-cell clones of uncertain significance (T-CUS). Although these entities involve distinct cellular compartments, they share the presence of a detectable clonal population in the absence of overt hematologic malignancy and may, in a subset of individuals, precede clinically manifest lymphoid or plasma-cell neoplasia. The parallel between MBL, MGUS, and T-CUS illustrates a broader biological principle whereby persistent antigenic or immune stimulation may provide a selective environment for the emergence and maintenance of hematopoietic clones [17]. In the context of the present review, MGUS represents the most relevant example of this general paradigm because of its direct relationship with clonal plasma-cell expansion and monoclonal immunoglobulin production [17].

According to current models, genetic abnormalities often do not play the primary role in the development of initial clonal expansion. It is more likely that sustained antigen stimulation provides certain immune cell clones with a proliferative advantage that favors the accumulation of further genetic and epigenetic alterations. This process may thus establish a potential link between autoimmunity and the development of monoclonal gammopathies [17].

Importantly, chronic antigenic stimulation should be regarded primarily as a potential initiating or selective pressure rather than as a requirement for the continued maintenance of every established MGUS clone. Once a plasma-cell clone has acquired sufficient intrinsic survival advantages and has established supportive interactions with the bone-marrow microenvironment, its persistence and subsequent evolution may become progressively less dependent on the original antigenic stimulus. Genetic and epigenetic alterations, together with changes in immune surveillance and the marrow niche, may then become increasingly important determinants of clonal fitness and progression. However, no clinically defined threshold currently identifies the point at which an antigen-driven clone becomes functionally autonomous, and this transition is likely to be gradual and heterogeneous among patients [20].

Why chronic immune activation results in polyclonal hypergammaglobulinemia in most patients but monoclonal gammopathy in only a subset remains incompletely understood. The emergence of MGUS probably requires the convergence of several factors rather than chronic antigenic stimulation alone, including age-related changes in immune surveillance, inherited susceptibility, acquired genetic or epigenetic alterations, persistent microbial or viral antigen exposure, and a bone-marrow microenvironment permissive for clonal plasma-cell survival [21,22,23]. Chronic infections such as HCV and HIV provide clinically relevant examples of prolonged antigenic stimulation associated with monoclonal gammopathy, but neither infection nor autoimmunity alone is sufficient to explain clonal selection [9]. Thus, the transition from polyclonal immune activation to MGUS is best regarded as a multistep process in which environmental and inflammatory pressures interact with clone-intrinsic and host-related susceptibility factors.

### 2.2. The Background of Monoclonal Cell Proliferation

#### 2.2.1. Activation of Follicular T-Helper Cells

Follicular T-helper (Tfh) cells are key regulators of humoral immunity and contribute to germinal-center formation, B-cell affinity maturation, and plasma-cell differentiation [24]. Tfh cells develop through the Bcl-6 transcription factor and interact with B cells via various cell-surface proteins, such as CXCR5, ICOS, PD-1, and CD40 ligand. Upon activation, Tfh cells produce IL-21, which promotes plasma-cell differentiation. Increased Tfh-cell activity has been reported in several autoimmune diseases, including RA, SLE, primary SS, and ANCA-associated vasculitides, and may contribute to sustained autoreactive B-cell responses [25,26]. Persistent Tfh-mediated B-cell stimulation may promote the survival of autoreactive clones and contribute to dysregulated immunoglobulin production. Dysregulation of Tfh-cell number and function has been implicated in several autoimmune diseases, including SLE, RA, and primary Sjögren’s syndrome, where enhanced Tfh-mediated B-cell help may contribute to persistent germinal-center activity, autoantibody production, and the survival of autoreactive B-cell clones [24,25,26]. The function of Tfh-cell activity may also be relevant to hematologic malignancies. In some Tfh-cell lymphomas, neoplastic Tfh cells retain B-cell helper functions, which may contribute to plasma-cell activation and hypergammaglobulinemia [27].

#### 2.2.2. Dynamic Disruptions in the Germinal Center

Germinal centers are the most important anatomical and functional units of the humoral immune response, serving as sites for B cell proliferation, somatic hypermutation, affinity maturation, and isotype switching [28,29,30,31,32,33]. Under normal circumstances, germinal center reactions are regulated by strict selection mechanisms, with follicular regulatory T cells (Tfr) playing a key role in preventing the survival and expansion of autoreactive B cell clones. If these regulatory mechanisms are compromised, the likelihood of survival for autoreactive or potentially malignant B cell clones increases. Disruption of germinal center selection can thus create an environment that promotes the development of monoclonal proliferation and subsequent malignant transformation [28,29,30].

#### 2.2.3. Common Cytokine Profile: IL-6, BAFF, and APRIL

Autoimmune disorders and plasma cell neoplasms share numerous common cytokine-signaling pathways. Among these, the BAFF-APRIL system and IL-6 signaling are of particular importance [10,11].

BAFF and APRIL are members of the TNF family: they play a fundamental role in B cell survival, proliferation, and differentiation. Abnormally elevated BAFF levels are detected in numerous autoimmune diseases (e.g., RA, SS, SLE) [34]. BAFF and APRIL are key regulators of B-cell and plasma-cell survival and may provide an important mechanistic link between chronic autoimmune activation and persistence of plasma-cell populations [34,35,36]. In myeloma cells, signaling through BAFF- and APRIL-binding receptors, including BCMA and TACI, can activate NF-κB, PI3K/AKT, and MAPK pathways and promote the expression of anti-apoptotic proteins, thereby enhancing plasma-cell survival [37].

IL-6 represents an important component of the bone-marrow cytokine network and can promote plasma-cell survival and proliferation through activation of the JAK/STAT3 pathway. Constitutive or sustained STAT3 activation enhances anti-apoptotic signaling and has been implicated in the persistence and progression of malignant plasma-cell populations [38,39,40].

#### 2.2.4. Links Between Chronic Inflammation and Oncogenesis: NF-κB and STAT3

Chronic inflammation is not merely a consequence of tumor development but can actively contribute to tumorigenesis [41]. One of the central regulators of this process is the NF-κB signaling pathway: its activation is triggered by inflammatory cytokines, infections, and various environmental factors. NF-κB enhances the transcription of numerous genes involved in cell cycle regulation, proliferation, and the inhibition of apoptosis [41,42].

Equally important is the STAT3 pathway, which links chronic inflammation and tumor formation primarily through IL-6-dependent activation. STAT3 activation enhances cell survival, promotes angiogenesis, and may contribute to the development of immune evasion and drug resistance [38,41].

**Figure 1:**
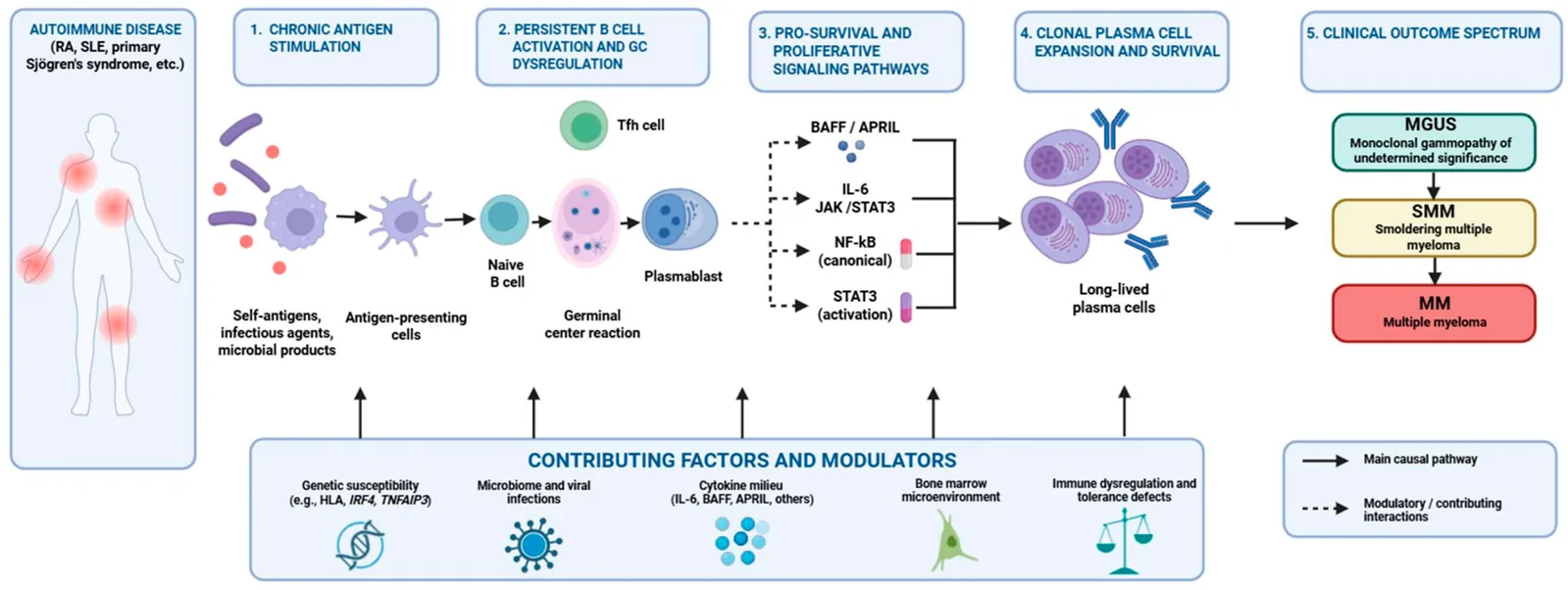
Shared pathogenetic mechanisms linking autoimmunity and monoclonal plasma-cell proliferation. Chronic antigen stimulation and persistent B-cell activation may promote germinal-center dysregulation, enhanced T follicular helper (Tfh)-cell activity, and sustained activation of pro-survival signaling pathways. The BAFF/APRIL system, IL-6/JAK/STAT3 signaling, NF-κB activation, and interactions with the bone marrow microenvironment may collectively support the survival and expansion of clonal plasma-cell populations. Genetic and epigenetic factors, immune microenvironmental changes, sustained B-cell receptor signaling, aging, immune senescence, and environmental triggers may further modulate this process. These interconnected mechanisms provide a biologically plausible link between chronic autoimmune/immune-mediated activation and the development of monoclonal gammopathy of undetermined significance (MGUS), which may remain stable or, in a subset of patients, progress to smoldering multiple myeloma (SMM) and ultimately multiple myeloma (MM). Figure was partly created with https://biorender.com/ (accessed on 4 October 2026). (Created in BioRender. Sipos, F. (2026) https://BioRender.com/7dd7dup. Created in BioRender. Sipos, F. (2026) https://BioRender.com/m0v4imy. Created in BioRender. Sipos, F. (2026) https://BioRender.com/bg9l15r).

Overall, chronic antigen stimulation, germinal-center dysregulation, increased Tfh cell activity, and IL-6-, BAFF-, APRIL-, NF-κB-, and STAT3-mediated signaling may form an interconnected biological network capable of promoting persistent B cell activation and supporting the survival and expansion of plasma-cell populations (Figure 1). Antigen-specific monoclonal immunoglobulins identified in subsets of patients provide additional support for an antigen-driven model in selected cases; however, studies of paraprotein antigen specificity have yielded heterogeneous results and have not consistently supported a universal causal role for chronic antigenic stimulation in MGUS or MM [18,19]. Importantly, single-cell transcriptomic studies have demonstrated that alterations in the bone marrow immune microenvironment are already detectable in precursor plasma-cell disorders and evolve across the MGUS–SMM–MM disease continuum, further supporting the contribution of immune dysregulation to clonal plasma-cell evolution [43,44]. Thus, the available mechanistic evidence supports a biologically plausible link between chronic autoimmune/immune-mediated activation and clonal plasma-cell proliferation but does not establish a single causal pathway applicable to all patients. The clinical relevance of these mechanisms is also likely to vary across individual autoimmune conditions; therefore, the following section examines the strength and nature of the associations supported by epidemiological and clinical data.

## 3. Epidemiological and Clinical Correlations

The development of monoclonal gammopathy has long been recognized across a broad spectrum of autoimmune diseases [45]; complex, abnormal immunoregulatory and inflammatory mechanisms are presumed to underlie this phenomenon. However, based on the available data, the strength and clinical significance of this correlation vary considerably among the different clinical conditions. Therefore, epidemiological and clinical evidence should be evaluated not merely on a disease-by-disease basis, but rather in terms of how much more common monoclonal gammopathy is in a given disease, what its prognostic significance is, and whether there is a genuine pathogenetic link underlying it.

### 3.1. The Relationship Between RA, MGUS, and MM

A large-scale study based on the U.S. National Inpatient Sample (NIS) database showed a significant association between RA and MGUS (OR 2.04, 95% CI 1.80–2.32), whereas no significant association was observed between RA and MM [46]. This association is supported by a subsequent systematic review and meta-analysis, which found an approximately twofold increased risk of monoclonal gammopathy in patients with RA compared with the general population [47]. However, the absence of a similarly increased risk of MM suggests that the relationship between chronic rheumatic inflammation, MGUS development, and subsequent malignant progression is not straightforward.

Significant differences are also observed in the risk of progression. Steiner et al. reported that the risk of progression from MGUS to multiple myeloma was higher in patients with certain non-antibody-mediated rheumatologic diseases, whereas no significant increase was observed in patients with antibody-mediated rheumatic diseases such as rheumatoid arthritis [48]. In contrast, a large population-based Swedish study including 19,303 patients with MGUS found that a history of autoimmune disease was associated with a lower risk of progression to multiple myeloma (HR 0.83, 95% CI 0.73–0.94) and other lymphoproliferative disorders (HR 0.84, 95% CI 0.75–0.94) compared with MGUS patients without autoimmune disease [49]. Thus, the available evidence does not support a uniform effect of autoimmune disease on MGUS progression and suggests that the impact may depend on the specific autoimmune or inflammatory phenotype.

In RA, particularly during treatment with anti-TNF agents (adalimumab, etanercept, and infliximab), the development of MGUS has been reported in several cases [50,51,52]. This phenomenon may be due to alterations in antitumor immune surveillance and reorganization of the cytokine network. In the majority of reported cases, MGUS did not progress to multiple myeloma. According to some reports, the monoclonal component appeared within 9–16 months after the start of treatment and then partially or even completely regressed following the discontinuation of biologic therapy.

Limited data are available on the role of methotrexate and JAK inhibitors. Based on current knowledge, effective inflammation control may mitigate chronic immune stimulation; however, in cases of prolonged immunosuppression, regular screening for malignant diseases is of paramount importance [50,51,52].

### 3.2. The Prevalence of MGUS in SLE

One of the defining immunological characteristics of SLE is marked B cell hyperactivity. In a cohort study involving 1083 patients, monoclonal gammopathy was detected in 5.4% of patients, which significantly exceeds the prevalence observed in the general population [53]. The most common isotype was IgG (2.95%), followed by IgM (1.29%) and IgA (1.1%) monoclonal gammopathies. These findings are consistent with a systematic review and meta-analysis demonstrating an approximately fourfold increased risk of monoclonal gammopathy in patients with SLE compared with the general population [47].

Although monoclonal gammopathy is often benign, recent findings suggest that malignancy and mortality may be increased among patients with SLE and a monoclonal component [53,54]. In a retrospective cohort of 1039 patients with SLE, malignancies were diagnosed in 8 of 23 patients with monoclonal gammopathy (34.8%), compared with 82 of 1016 patients without monoclonal gammopathy (8.1%), over a median follow-up of 11 years (interquartile range, 6–19 years) [54]. However, these findings should be interpreted cautiously because the study was retrospective and conducted at tertiary referral departments of a single university hospital, potentially introducing referral and selection bias and limiting the generalizability of the results.

B cells play a central role in the pathogenesis of lupus nephritis through their production of autoantibodies, their antigen-presenting function, and their cytokine production. Sustained B cell activation and proliferation may increase the likelihood of genomic instability and clonal expansion, which could provide a theoretical basis for the higher incidence of MGUS and MM. Accordingly, modern therapeutic approaches focus on B cell-depleting therapies (rituximab, obinutuzumab) and the inhibition of BAFF/APRIL signaling [55,56]. In addition, targeted inhibition of IFN-α signaling (anifrolumab) may also represent a promising treatment alternative [57].

### 3.3. Sjögren’s Syndrome and Monoclonal Gammopathy

Among autoimmune diseases, primary Söjgren’s syndrome (SS) shows one of the sötrongest associations with B-cell lymphoproliferative disorders and monoclonal gammopathy. The reported prevalence of monoclonal gammopathy varies considerably across cohorts, ranging from 7.4% in a cohort of 352 patients to approximately 22% in other series [10,58,59], likely reflecting differences in patient selection, disease characteristics, and detection methods. A systematic review and meta-analysis further demonstrated that, among autoimmune inflammatory rheumatic diseases, SS was associated with the highest relative risk of monoclonal gammopathy compared with the general population (OR 4.51, 95% CI 3.39–5.74) [47]. Importantly, the presence of a monoclonal component has been associated with higher disease activity, an increased risk of lymphoma and other hematologic malignancies, and poorer outcomes, supporting its potential prognostic relevance [10,58,60]. IgG-kappa has been reported as a common monoclonal component [59].

Older studies also reported particularly high frequencies of monoclonal gammopathy in patients described as having HCV-associated Sjögren’s syndrome, reaching approximately 52% in one series [58]. However, these historical data require careful interpretation in light of current classification criteria. Under the 2016 ACR/EULAR classification criteria, active hepatitis C infection (with positive PCR) is an exclusion criterion for primary Sjögren’s syndrome; consequently, HCV-associated sicca manifestations should be considered separately when interpreting historical primary SS cohorts [61]. Differences in case definition and the inclusion of HCV-associated sicca syndromes in older cohorts may therefore partly account for the wide variation in reported monoclonal gammopathy prevalence across studies of Sjögren’s syndrome [58,61].

The B cell hyperactivity characteristic of SS is primarily driven by BAFF excess, chronic antigen stimulation, and reduced immunotolerance. These processes often lead to the development of mixed cryoglobulinemia. The distinction between cryoglobulinemia subtypes is particularly relevant when interpreting clonality. Type I cryoglobulinemia consists of a monoclonal immunoglobulin and is typically associated with an underlying clonal B-cell or plasma-cell disorder. In contrast, type II mixed cryoglobulinemia contains polyclonal IgG together with a monoclonal, most commonly IgM, rheumatoid factor and is frequently associated with chronic HCV infection as well as autoimmune or lymphoproliferative disorders. Type III mixed cryoglobulinemia contains polyclonal immunoglobulins and therefore does not, by itself, indicate an underlying monoclonal gammopathy. This distinction is important because the presence of cryoglobulinemia does not uniformly imply the same degree or biological meaning of B-cell clonality [62]. Type II mixed cryoglobulinemia may cause vasculitis and organ damage through the formation and deposition of immune complexes containing monoclonal IgM with rheumatoid-factor activity. Thus, the hypergammaglobulinemia and monoclonal gammopathy observed in SS may correspond to different manifestations of the same pathological spectrum of B-cell activation [59].

### 3.4. Polyclonal and Monoclonal Immune Activation in Autoimmune Liver Diseases

A classic immunological feature of autoimmune hepatitis (AIH) is marked polyclonal hypergammaglobulinemia, which reflects an imbalance in the immunological equilibrium between regulatory T cells and B cells. Although B cells play a decisive role in the pathogenesis of the disease, AIH is less frequently associated with monoclonal expansion than other systemic autoimmune diseases [63,64].

In addition to producing autoantibodies, B cells also contribute to the maintenance of chronic liver inflammation through their antigen-presenting and cytokine-producing functions. Nevertheless, based on available data, the association between AIH and plasma cell dyscrasias is less pronounced than, for example, in SLE or SS [63,64].

In primary biliary cholangitis (PBC), however, associations with MM have been reported. Some case reports—including observations of patients with lambda-light-chain myeloma—suggest that persistent liver inflammation and impaired immunological tolerance may favor the development of clonal B-cell expansion [65]. However, the epidemiological significance of this association is not yet known, so further studies are needed to clarify whether PBC can be considered an independent risk factor for plasma cell neoplasms.

### 3.5. Psoriasis, Multiple Myeloma, and the Role of the Inflammatory Cytokine Environment

Psoriasis is a chronic, systemic inflammatory disease characterized by increased production of numerous proinflammatory cytokines—including TNF-α, IL-6, IL-17, and IL-23. Since IL-6 plays a key role in the survival and proliferation of plasma cells, this fact could theoretically contribute to the development of monoclonal gammopathies. It is known that a persistently altered cytokine profile is observed in MGUS and MM, which in principle may provide a common pathogenetic basis with chronic inflammatory diseases [13,66]. However, there is currently no evidence of a clear causal relationship between the development of psoriasis and MM. Although large population-based studies have reported an association between psoriasis and an increased incidence of several malignancies, including MM, these epidemiological observations do not establish a direct causal relationship between chronic psoriatic inflammation and plasma-cell neoplasia [67]. In fact, another multicountry psoriasis cohort study from 2026 shows that the association with MM is not the same in all populations [68].

However, the potential impact of biologic therapies warrants special attention, as the development of MGUS has been reported in patients with psoriasis receiving anti-TNF therapy [50,51,52]. Several years typically elapse between the development of the monoclonal component and the initiation of therapy, suggesting a provocative role for long-term immunomodulation.

### 3.6. Other Conditions Associated with Immune Dysregulation and Monoclonal Gammopathy

In addition to classical autoimmune diseases, several infectious, inflammatory, and immune-dysregulatory conditions are relevant to the relationship between chronic immune stimulation and monoclonal gammopathy.

#### 3.6.1. Viral Infections and Monoclonal Gammopathy

Monoclonal gammopathy can develop in the course of several chronic viral infections. The underlying mechanisms primarily include persistent antigen stimulation, chronic B cell activation, and, in certain infections, virus-specific alterations in immune regulation. The association is particularly well documented in chronic HCV and HIV infection. In a prospective study of patients with chronic liver disease, monoclonal gammopathy was detected in 11% of HCV-positive patients compared with 1% of HCV-negative controls [69]. Similarly, an increased prevalence of monoclonal gammopathy has been reported in people living with HIV, often occurring at a younger age than in the general population [70,71]. These observations support the concept that persistent viral antigenic stimulation may contribute to the emergence or maintenance of clonal B cell or plasma-cell populations [72,73,74].

If the onset of monoclonal gammopathy is closely associated with a viral infection, regression or stabilization of the monoclonal component is sometimes observed following successful antiviral treatment. Thus, in chronic HCV infection, direct-acting antiviral agents may not only result in viral elimination but may also sometimes lead to sustained hematologic remission [72,73,74]. Progression of MGUS appears to be rare among patients receiving antiviral treatment, and a more favorable clinical outcome is observed even in the presence of multiple myeloma [73,74].

#### 3.6.2. IgG4-Related Diseases and Monoclonal Gammopathy

IgG4-related disease (IgG4-RD) is a systemic fibroinflammatory condition that can affect multiple organs, most commonly the pancreas, kidneys, lacrimal and salivary glands, and the retroperitoneum [75]. The disease is characterized by tumor-like organ lesions, lymphoplasmacytic infiltration, and significantly elevated serum IgG4 levels [75].

According to previous studies, the incidence of malignant tumors may be increased among patients with IgG4-RD [76]. However, the co-occurrence of IgG4-RD and MGUS is rare: a total of three cases has been reported to date [77,78]. All patients were over 60 years of age. It has been suggested that the persistent inflammatory microenvironment characteristic of IgG4-RD may promote the development of clonal plasma cell expansion.

#### 3.6.3. Monoclonal Gammopathy in Other Autoimmune, Immune-Mediated, and Autoinflammatory Conditions

Monoclonal gammopathy can also occur in other immune-mediated conditions, such as systemic sclerosis (SSc), idiopathic inflammatory myopathies—particularly dermatomyositis (DM) and necrotizing autoimmune myopathy (NAM)—polymyalgia rheumatica (PMR), ankylosing spondylitis (AS), undifferentiated autoimmune syndrome (UCTD), and various vasculitides. The presence of a monoclonal component has also been described in neurological and dermatological conditions, such as bullous pemphigoid, chronic inflammatory demyelinating polyneuropathy (CIDP), multiple sclerosis, and myasthenia gravis [9,13,47,79,80,81]. In most of these associations, however, it remains unclear whether monoclonality is merely a concomitant phenomenon, a consequence of chronic immune activation, or whether it plays a genuine pathogenetic role. Castleman disease and TAFRO syndrome also merit consideration in the differential diagnosis of systemic inflammatory disorders accompanied by immunoglobulin abnormalities. However, unlike classical monoclonal gammopathies, idiopathic multicentric Castleman disease is typically associated with cytokine-driven polyclonal hypergammaglobulinemia, whereas a prominent monoclonal protein is not a characteristic feature of TAFRO syndrome [82]. These entities should therefore not be regarded as typical MGCS disorders, although their clinical overlap with inflammatory, lymphoproliferative, and plasma-cell disorders may create diagnostic challenges [82]. Castleman disease is also relevant to the differential diagnosis of POEMS syndrome and constitutes one of its established major diagnostic criteria [83]. Among autoinflammatory diseases, Schnitzler syndrome deserves special attention, as monoclonal gammopathy is an integral part of the clinical phenotype in this condition and serves as a good example of how there may be a genuine biological link between persistent inflammatory activity and clonal immunoglobulin production [84]. This observation suggests that, under certain circumstances, chronic inflammation may not merely be associated with, but may actually contribute to, the development or persistence of monoclonal gammopathy.

Importantly, progression risk and overall prognosis should not be considered synonymous. In a large Swedish population-based study, a previous autoimmune disease was associated with impaired overall survival among patients with MGUS, despite other population-based data suggesting that autoimmune disease may be associated with a lower risk of malignant progression [85].

Above all, recent population-based screening data have challenged the assumption that autoimmune diseases are uniformly associated with an increased prevalence of MGUS. In the iStopMM study, which systematically screened more than 75,000 individuals, a history of autoimmune disease was not significantly associated with screen-detected MGUS after adjustment for age and sex, despite a clear association with previously clinically diagnosed MGUS [6,13]. This discrepancy suggests that at least part of the association reported in earlier clinically ascertained cohorts may reflect increased diagnostic activity and ascertainment bias.

Overall, the epidemiological and clinical data available for individual diseases thus present a heterogeneous picture. An increase in the incidence of monoclonal gammopathy does not in itself prove a causal relationship: the same observation may reflect persistent immune activation and the resulting clonal expansion, or it may simply be a coincidence arising from age, genetic factors, or increased diagnostic activity. Distinguishing among these possibilities is therefore central to the interpretation of monoclonal gammopathy in autoimmune diseases. The principal epidemiological and clinical features of monoclonal gammopathy across selected autoimmune and immune-mediated conditions are summarized in Table 1.

## 4. Interpretation of Monoclonal Gammopathy in Autoimmune Diseases

The relationship between monoclonal gammopathy and autoimmune or immune-mediated diseases is receiving increasing attention; however, the nature of the association between these two phenomena remains unclear. Theoretically, it is possible that monoclonal gammopathy may serve as a marker of autoimmune processes, and it may also develop as a consequence of them; it may be a pathogenic factor, or in certain cases, it may simply be a coincidental association [4,9].

### 4.1. Monoclonal Gammopathy as a Marker

According to one of the most widely accepted hypotheses, monoclonal gammopathy can be regarded as a marker of chronic antigen stimulation and sustained immune activation. In many autoimmune diseases, there is continuous activation and selection of B cells, which can lead to clonal expansion over the long term. The laboratory manifestation of this may be the detection of M-protein or an increase in its concentration [9].

A particularly well-studied example is primary SS, in which the presence of monoclonal gammopathy is associated with increased disease activity and a higher risk of developing hematologic malignancies [10]. Based on these findings, paraproteinemia is, in certain cases, not merely a concomitant phenomenon but can also be interpreted as an indirect biomarker of immune system activity.

It has also been suggested that monoclonal gammopathy may serve as a useful marker for monitoring disease activity and prognosis in conditions with pathomechanisms similar to those of RA or SS, although its clinical applicability requires further investigation [9,10].

### 4.2. Monoclonal Gammopathy as a Consequence

According to another hypothesis, monoclonal gammopathy develops as a consequence of autoimmune inflammation. The continuous B cell activation, increased cell proliferation, and inflammatory microenvironment present during chronic inflammation may promote the accumulation of genetic and epigenetic alterations, which can ultimately lead to the formation of a clonal plasma cell population [5,9].

IL-6 is an important regulator of plasma-cell survival and proliferation and exerts many of its effects through activation of the JAK/STAT3 signaling pathway [38]. Prolonged cytokine stimulation may promote the persistence and expansion of clonal cell lines and contribute to the progression of monoclonal gammopathy [37,38].

Epidemiological observations in autoimmune diseases, including SLE, are compatible with a potential link between persistent immune activation and clonal plasma-cell expansion; however, the available evidence does not establish a direct causal relationship between autoimmune inflammation and progression to MM. Moreover, increased occurrence of monoclonal gammopathy, increased overall malignancy risk, and progression of established MGUS to MM represent distinct clinical outcomes and should not be interpreted interchangeably [11,47,48,49,53,54].

In principle, another possible explanation could also be considered in the context of immunosuppressive therapies, as prolonged immunosuppression may reduce tumor immunological control, thereby promoting the persistence of premalignant clonal cell lines, although only limited evidence is available regarding this [9,11].

The link between inflammation and clonal plasma cell proliferation is further supported by studies that have demonstrated reduced immunoglobulin sialylation in MGUS and MM. This observation suggests that chronic inflammation may not only be a consequence but also a potential contributor to the development and progression of clonal plasma cell disorders [13].

### 4.3. Monoclonal Gammopathy as a Pathogenetic Factor

In recent years, the concept of monoclonal gammopathy of rheumatologic significance (MGRhS) has emerged, encompassing conditions in which a clonal B cell or plasma-cell population that does not meet the criteria for overt hematologic malignancy contributes directly to systemic manifestations or organ damage in rheumatologic diseases [12]. MGRhS can be viewed within the broader framework of monoclonal gammopathy of clinical significance (MGCS), which recognizes that even small, otherwise premalignant B cell or plasma-cell clones may have clinically relevant consequences through the pathogenic effects of their monoclonal immunoglobulins [4,5,12]. A well-established example of this paradigm is monoclonal gammopathy of renal significance (MGRS), in which a clonal proliferative disorder produces a nephrotoxic monoclonal immunoglobulin despite not meeting the hematologic criteria for treatment of an overt malignancy [86]. Similarly, monoclonal gammopathy of neurological significance (MGNS) illustrates that a monoclonal gammopathy without overt hematologic malignancy may be associated with clinically significant neurological dysfunction, particularly peripheral neuropathy [87].

AL amyloidosis provides a paradigmatic example of the pathogenetic potential of a monoclonal protein. In this disorder, an often relatively small plasma-cell clone produces misfolded immunoglobulin light chains that form amyloid fibrils and deposit in tissues, resulting in potentially severe cardiac, renal, neurological, hepatic, or other organ dysfunction [88]. Thus, the clinical impact of the clone is determined primarily by the pathogenic properties of the secreted immunoglobulin rather than by tumor burden alone. Conceptually, AL amyloidosis therefore strongly illustrates the principle underlying MGCS, although it is generally regarded as a well-established disease entity in its own right [89].

POEMS syndrome provides another multisystem example in which a clonal plasma-cell disorder is associated with clinically significant organ dysfunction that cannot be explained by tumor burden alone. The syndrome is characterized by polyneuropathy and an underlying monoclonal plasma-cell disorder, usually lambda-restricted, together with systemic manifestations driven in part by markedly increased pro-inflammatory and angiogenic mediators, particularly vascular endothelial growth factor. POEMS therefore further illustrates how a relatively limited clonal plasma-cell population may produce disproportionate multisystem clinical consequences [89].

These concepts collectively emphasize that the clinical significance of monoclonal gammopathy is determined not only by the size or malignant potential of the underlying clone, but also by its capacity to cause organ damage. The principal organ- and disease-specific concepts within the broader spectrum of clinically significant monoclonal gammopathies are summarized in Table 2. Although these entities differ in terminology and scope, they share the concept that the clinical relevance of a clone is determined not solely by its size or malignant potential but also by its capacity to cause organ-specific or systemic disease.

### 4.4. Monoclonal Gammopathy as a Coincidental Finding

However, it cannot be ruled out that some of the observed associations are the result of random coincidence. The prevalence of MGUS increases significantly with advancing age, reaching 3–5% in the population over 50 years of age, and is even more common in older age groups [9]. Numerous autoimmune diseases also occur primarily in middle-aged and older adults; therefore, age may be an important predisposing factor.

Age-related clonal hematopoiesis provides an additional biological context for interpreting apparently coincidental associations between monoclonal gammopathy and autoimmune disease [90]. Clonal hematopoiesis of indeterminate potential (CHIP), MGUS, and monoclonal B-cell lymphocytosis all become increasingly prevalent with advancing age and represent distinct manifestations of age-associated clonal expansion within the hematopoietic system. Moreover, immunosenescence and inflammageing may alter immune surveillance and the bone-marrow microenvironment, thereby favoring the emergence or persistence of hematopoietic clones. Thus, age may act not merely as a statistical confounder but as a shared biological background promoting both immune dysregulation and clonality. Importantly, however, CHIP should not be regarded as a direct precursor or established cause of MGUS; rather, it illustrates the broader age-related propensity toward clonal hematopoiesis [90].

The results of epidemiological studies may also be influenced by increased diagnostic activity, as patients with autoimmune diseases generally undergo regular laboratory testing, making it more likely that even clinically insignificant paraproteinemia will be detected [9,13]. Additional confounding factors may include genetic differences, the presence of comorbidities, and the use of immunosuppressive or biologic therapies. For this reason, epidemiological associations alone are insufficient to prove a causal relationship [9].

**Figure 2:**
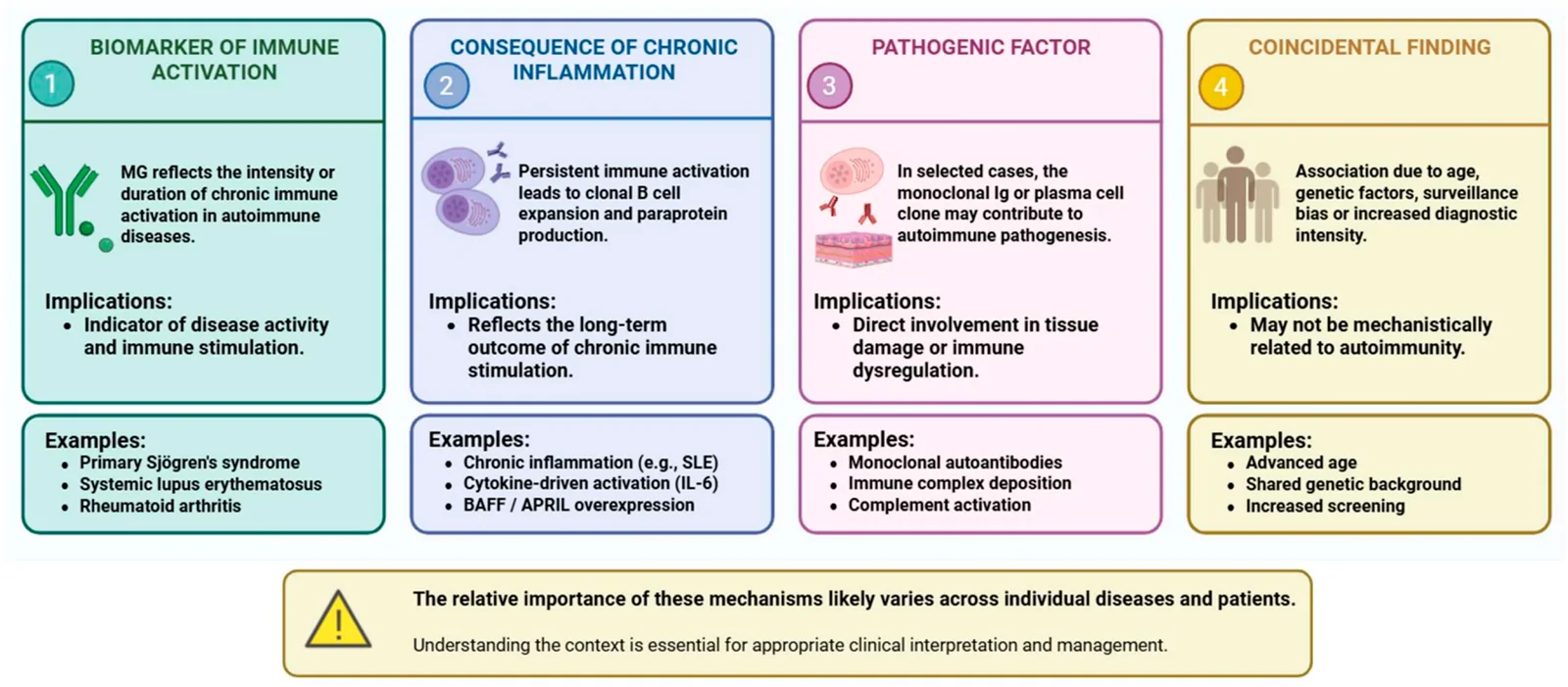
Four possible roles of monoclonal gammopathy in autoimmune diseases. The association between monoclonal gammopathy and autoimmune/immune-mediated diseases can be interpreted according to four, potentially overlapping models. First, monoclonal gammopathy may serve as a marker of immune activation, reflecting persistent B cell stimulation and, in some diseases, increased disease activity or risk of lymphoproliferative complications. Second, it may represent a consequence of chronic immune activation, whereby sustained inflammation, B cell stimulation, and cytokine-mediated survival signals promote clonal expansion. Third, in selected conditions, the monoclonal immunoglobulin or the underlying clonal B cell/plasma-cell population may act as a pathogenetic factor, directly contributing to immune-mediated manifestations or organ damage. Finally, monoclonal gammopathy may represent a coincidental finding, particularly in older individuals or in the presence of shared genetic factors or increased diagnostic surveillance. These mechanisms are not mutually exclusive, and their relative contribution is likely to vary among diseases and individual patients. Figure was partly created with https://biorender.com/ (accessed on 4 October 2026). (Created in BioRender. Sipos, F. (2026) https://BioRender.com/9lm4lwh. Created in BioRender. Sipos, F. (2026) https://BioRender.com/avqd1ec).

In summary, based on current evidence, the relationship between monoclonal gammopathy and autoimmune diseases is quite heterogeneous, and clarifying it requires further prospective clinical and translational studies (Figure 2).

These four interpretative models should not be regarded as fixed categories throughout the clinical course. Their relative importance may change over time within the same patient. An MGUS clone that initially emerges in the setting of chronic immune stimulation may subsequently acquire biological features that permit increasingly autonomous persistence and evolution. Once MGUS is established, its hematologic behavior should therefore be assessed according to clone-specific risk factors rather than inferred solely from the activity or treatment response of the underlying autoimmune disease. If progression to smoldering or symptomatic multiple myeloma occurs, management is determined by the biology and clinical criteria of the plasma-cell disorder rather than by its presumed initial relationship with autoimmunity. To facilitate a critical interpretation of these potentially overlapping relationships, the principal findings supporting or arguing against each of the four proposed models are summarized in Table 3.

## 5. Clinical Considerations

Based on the above, the detection of monoclonal gammopathy in autoimmune diseases cannot, in and of itself, be interpreted as a uniform prognostic or pathogenetic marker. From a clinical practice perspective, the most important questions are therefore the following: In which patients is targeted screening for monoclonal gammopathy warranted? How should confirmed MGUS be monitored? Is it necessary to modify the treatment of the autoimmune disease?

### 5.1. Who Should Be Screened and When?

Currently, there is no clear evidence to justify routine screening for monoclonal gammopathy among patients with autoimmune or immune-mediated diseases; however, risk-based, targeted monitoring may be warranted in certain clinical conditions. In particular, high prevalences of paraproteinemia and monoclonal gammopathy have been observed in primary Sjögren’s syndrome, which is associated with an increased risk of lymphoma and multiple myeloma [9,10,58]. Similarly, targeted screening may be considered for SLE patients who present with unexplained hypergammaglobulinemia, cytopenia, kidney damage, or unusual systemic symptoms [53,54].

### 5.2. Monitoring MGUS in Autoimmune Diseases—Does It Differ from the Standard Algorithm?

Current international guidelines do not distinguish between the monitoring of MGUS associated with autoimmune diseases and that of sporadic cases. Monitoring continues to be based on M-protein levels, immunoglobulin isotype, free light chain ratio, and the presence of any organ damage [1,3,91,92].

At the same time, a growing body of evidence suggests that an autoimmune background may, in certain cases, modify the risk of progression. Importantly, the effect of autoimmune disease on the natural history of MGUS appears to be heterogeneous. Steiner et al. reported that certain rheumatologic diseases, particularly non-antibody-mediated inflammatory conditions, may be associated with an increased risk of progression to multiple myeloma [48]. Conversely, a large population-based study found a lower risk of progression to multiple myeloma and other lymphoproliferative disorders among MGUS patients with a history of autoimmune disease [49]. These apparently divergent findings indicate that autoimmune disease should not currently be considered an independent, uniform risk factor for MGUS progression. Until more disease-specific evidence becomes available, MGUS monitoring should therefore continue to follow established risk-stratification criteria rather than being intensified solely on the basis of an autoimmune diagnosis.

Importantly, MGUS risk assessment should also be regarded as a dynamic process rather than a one-time evaluation at diagnosis. In a longitudinal study of patients with MGUS, evolving changes in serum immune markers, including increasing M-protein and involved free light-chain levels as well as increasing immunoparesis, were associated with subsequent progression to multiple myeloma [93]. Moreover, among patients who progressed and had a high-risk score before multiple myeloma diagnosis, a substantial proportion had previously been classified as low or intermediate risk, supporting the concept that progression risk may evolve over time [93]. Consistent with these findings, a subsequent longitudinal analysis of 427 patients demonstrated that the Mayo2005, Sweden2014, and NCI2019 risk stratification models remained informative when repeatedly applied during follow-up and that migration to a higher-risk category was associated with an increased risk of progression [94]. Together, these findings support periodic reassessment of established MGUS risk factors during surveillance, particularly when clinical or laboratory parameters change.

A Chinese cohort study further clarified the considerations for follow-up of autoimmune patients with MGUS [79]. According to the authors, high baseline M-protein levels, weight loss, and a decrease in uninvolved immunoglobulins (uninvolved Ig) are independent prognostic factors for the development of hematologic malignancy. For patients with these risk factors, closer monitoring may be warranted, including more frequent M-protein testing during the first six months, bone marrow examination if necessary, and at least annual follow-up thereafter. The study confirmed that a high baseline M-protein concentration remains one of the most important predictive factors; however, in this cohort of patients with autoimmune diseases, the M-protein isotype alone did not prove to be an independent risk factor for hematologic malignant transformation [79].

Prospective cohort studies are needed in the future to determine whether different monitoring strategies can be associated with specific autoimmune phenotypes.

### 5.3. Therapeutic Implications

#### 5.3.1. The Effect of Immunosuppressive Treatments

Immunosuppressive and biological therapies used in the treatment of autoimmune diseases may, in theory, influence the formation and persistence of clonal plasma cell populations. Alterations in antitumor immune surveillance and changes in the cytokine environment may favor the persistence of premalignant clones, although only limited evidence is available to support this [9].

In several cases, the development of MGUS has been reported in RA and psoriasis following anti-TNF therapy; however, a causal relationship has not been established, and in most cases, no progression occurred [50,51,52]. Even fewer data are available regarding methotrexate, JAK inhibitors, and other targeted therapies.

However, certain B cell-targeted therapies—such as rituximab or the BAFF inhibitor belimumab—could theoretically reduce clonal B cell activation, although their effect on MGUS progression is not yet known [55,56,57].

An additional clinical consideration is that MGUS itself may be accompanied by impaired humoral immunity and an increased susceptibility to infection, which may be further compounded by immunosuppressive treatment of the underlying autoimmune disease [95,96]. Therefore, treatment should aim to control clinically significant autoimmune inflammation while avoiding unnecessary cumulative immunosuppression, particularly in patients with recurrent infections, immunoparesis, or other evidence of impaired immune function. Importantly, there is currently insufficient evidence that conventional DMARDs, glucocorticoids, or biologic therapies prevent the development of MGUS or halt its progression to multiple myeloma [48,97]. Accordingly, autoimmune disease control and MGUS surveillance should be regarded as related but distinct clinical objectives, with treatment individualized according to autoimmune disease activity, infection risk, and established hematologic risk factors for MGUS progression.

#### 5.3.2. Treatment of RA, SS, and SLE in the Presence of MGUS

Based on current data, the presence of MGUS alone does not constitute a contraindication to the use of modern immunomodulatory or biologic therapies. The therapeutic decision is primarily determined by the activity of the underlying disease and the patient’s general condition.

However, in cases of known MGUS, regular hematologic monitoring is recommended, especially if the patient is receiving long-term immunosuppressive therapy. In SS, the presence of paraproteinemia requires close attention, as it may be an early marker of lymphoma or other clonal B cell disorders [10,58].

In current clinical practice, a risk-based approach integrating hematological and immunological considerations is increasingly warranted, particularly in patients in whom paraproteinemia is accompanied by systemic manifestations or organ damage. Early multidisciplinary collaboration may facilitate the timely recognition of disease progression and the development of an appropriate therapeutic strategy. However, substantial knowledge gaps remain, particularly regarding disease-specific progression risk, the role of targeted screening, and the long-term effects of immunomodulatory therapy. Further molecular studies and well-designed prospective clinical investigations are therefore needed to address these unresolved questions and to refine evidence-based strategies for the management of monoclonal gammopathy in patients with autoimmune and immune-mediated diseases.

#### 5.3.3. Emerging B-Cell- and Plasma-Cell-Directed Therapies

Emerging B-cell- and plasma-cell-directed therapies add a further dimension to this therapeutic interface. CD19-directed CAR-T-cell therapies and bispecific antibodies targeting B-cell or plasma-cell antigens, including CD20 and BCMA, are increasingly being investigated in severe refractory autoimmune diseases and can induce profound depletion of pathogenic B-cell and, depending on the target, plasma-cell compartments [98,99]. These approaches are conceptually relevant to patients in whom autoimmunity and clonal B-cell/plasma-cell disorders coexist; however, they should not currently be regarded as treatment for otherwise asymptomatic MGUS. Moreover, profound B-cell or plasma-cell depletion may result in hypogammaglobulinemia and increased infection risk [98,100]. Whether such therapies can simultaneously modify autoimmune disease activity and the natural history of a coexisting premalignant clone remains an important question for future prospective studies.

## 6. Future Research Directions

Research into the relationship between monoclonal gammopathies and autoimmune diseases has made significant progress in recent years; however, many fundamental questions remain open and unanswered. One of the most important goals of future research is to determine what paraproteinemia actually represents: is it a marker of autoimmune processes, a consequence of them, or—in certain cases—even an active pathogenic factor?

Single-cell omics technologies offer new opportunities for the detailed characterization of clonal B cell and plasma-cell populations and their surrounding immune microenvironment. Single-cell RNA-sequencing studies have already identified substantial alterations in the bone marrow immune microenvironment across the spectrum from MGUS and SMM to overt MM, indicating that immune dysregulation is detectable at precursor stages and evolves during disease progression [43,44]. However, comparable longitudinal single-cell data specifically addressing autoimmune-associated MGUS remain scarce. Future studies integrating single-cell transcriptomics, B cell receptor repertoire analysis, and spatial transcriptomics may help determine whether chronic autoimmune stimulation generates distinct clonal and microenvironmental trajectories that influence the development and progression of MGUS.

Long-term prospective cohort studies are also of paramount importance. It is not yet precisely known which autoimmune conditions and immunological phenotypes are associated with the highest risk of progression. The conflicting observations from existing cohorts further emphasize the need for prospective, disease-specific studies [48,49]. Future investigations should determine whether differences in autoimmune phenotype, the type and duration of chronic antigenic stimulation, immunomodulatory treatment, and the molecular characteristics of the underlying plasma-cell clone account for the divergent effects of autoimmune disease on MGUS progression. Integrating longitudinal clinical, immunological, and molecular data may ultimately establish whether autoimmune-associated MGUS represents a biologically distinct subgroup with a different natural history and progression risk.

In the future, predictive models may become available that take into account clinical characteristics, cytokine profiles, genetic variations, and the molecular characteristics of clonal cells. A particularly promising area of research is the study of the BAFF/APRIL system. Since this signaling network plays a central role in both autoimmune diseases and plasma cell neoplasms, it is possible that BAFF-, APRIL-, or BCMA-targeted therapies could influence not only autoimmune activity but also clonal plasma cell expansion [34]. Future clinical trials may clarify whether these therapeutic strategies will be able to reduce the risk of MGUS progression.

A new research direction has emerged: the investigation of the role of PANoptosis, which involves the integrated regulation of programmed inflammatory cell death pathways, including pyroptosis, necroptosis, and apoptosis [80]. Findings from recent years underscore that these processes may play a decisive role in the link between chronic inflammation and tumorigenesis. Of particular interest is how dysregulation of PANoptosis affects the bone marrow microenvironment, plasma cell survival, and the early stages of myelomagenesis. Given that PANoptosis appears to play an increasingly important role in the pathomechanisms of autoimmune and autoinflammatory diseases, elucidating these processes may also represent a new link between autoimmunity and clonal plasma cell disorders.

## 7. Conclusions

Based on the available epidemiological, clinical, and pathogenetic data, the relationship between monoclonal gammopathy and autoimmune/immune-mediated diseases cannot be considered a uniform phenomenon. In certain conditions—particularly primary SS, SLE, and RA—chronic antigen stimulation, sustained B cell activation, and an inflammatory microenvironment likely contribute to the development and persistence of clonal plasma cell populations. In some cases, monoclonal gammopathy can be considered a biomarker of immune activation, while in others it appears because of such activation; sometimes it may be a pathogenic factor, and in certain patients, it may simply represent a coincidental association. However, the newly introduced MGCS and MGRhS concepts suggest that clonal B cell abnormalities may themselves contribute to the development of organ damage.

From a practical perspective, current evidence does not support routine screening for monoclonal gammopathy in all patients with autoimmune or immune-mediated diseases; rather, testing should be guided by the underlying disease and by clinical or laboratory features suggestive of a clonal disorder. Once MGUS is identified, surveillance should generally follow established hematologic risk-stratification criteria, with periodic reassessment rather than intensified monitoring solely because of the autoimmune diagnosis. Particular attention and multidisciplinary hematologic–immunologic evaluation are warranted when paraproteinemia is accompanied by unexplained cytopenias, renal dysfunction, systemic manifestations, or other evidence of organ damage. Future molecular and longitudinal studies are expected to enable more accurate identification of high-risk patients and the identification of new diagnostic and therapeutic targets.

## Figures and Tables

**Table 1:** Evidence summary of the association between monoclonal gammopathy and selected autoimmune/immune-mediated diseases.

Disease/Condition	Evidence for Association with Monoclonal Gammopathy	Representative Epidemiological Findings	Clinical/Prognostic Significance	Proposed Interpretation	Key References
**Rheumatoid arthritis (RA)**	Moderate	Significant association between RA and MGUS in a U.S. NIS study (OR 2.04, 95% CI 1.80–2.32); no significant association with MM in the same analysis	Available data on MGUS progression are heterogeneous; antibody-mediated rheumatic diseases, including RA, have not consistently been associated with increased progression risk	Chronic immune stimulation and B-cell activation; potential contribution of immunomodulatory treatment	[46,47,48,49,50,51,52]
**Systemic lupus erythematosus (SLE)**	Moderate–strong	MG detected in 5.4% (59/1083) of patients in an earlier cohort; meta-analytic data indicate an approximately fourfold higher occurrence of MG compared with controls	A recent retrospective cohort found a higher malignancy rate in SLE patients with MG than in those without MG (34.8% vs. 8.1%); prognostic evidence remains limited	Sustained B-cell activation, chronic antigenic stimulation, and clonal selection	[47,53,54]
**Primary Sjögren’s syndrome (pSS)**	Strong	MG prevalence varied from 7.4% (26/352) to approximately 22% (48/221) across cohorts; systematic review: OR 4.51 compared with controls/general population	Associated with higher disease activity and increased risk of lymphoproliferative/hematologic malignancy; may have prognostic relevance	Persistent B-cell activation, BAFF-driven B-cell survival, chronic antigenic stimulation, and emergence of clonal B-cell populations	[10,47,58,59,60]
**Autoimmune hepatitis (AIH)**	Limited	Monoclonal expansion appears to be less frequent than in systemic autoimmune diseases; robust prevalence estimates are lacking	Clinical and prognostic significance remains uncertain	Predominantly polyclonal immune activation; monoclonal expansion may occur in selected cases	[63,64]
**Primary biliary cholangitis (PBC)**	Very limited	Evidence is based predominantly on individual reports; reliable epidemiological estimates are lacking	Clinical significance of the association remains uncertain	Chronic immune stimulation may occasionally promote clonal B-cell/plasma-cell expansion	[65]
**Psoriasis**	Limited/inconsistent	Epidemiological findings regarding MG/MM are heterogeneous; MGUS has also been reported during anti-TNF therapy	A causal relationship between psoriasis and MG has not been established	Chronic cytokine-mediated inflammation and/or treatment-related immune modulation may contribute in selected patients	[50,51,52,66,67,68]
**Chronic hepatitis C virus (HCV) infection**	Moderate	Monoclonal bands were detected in 11% of HCV-positive versus 1% of HCV-negative patients with chronic liver disease in one prospective study	Monoclonal gammopathy may regress or stabilize following successful antiviral therapy in selected patients	Persistent viral antigen-driven B-cell stimulation and clonal expansion	[69,72,73,74]
**Human immunodeficiency virus (HIV) infection**	Moderate	Increased occurrence of monoclonal gammopathy has been reported, often at a younger age than in the general population	Clinical course is variable; monoclonal proteins may be transient or persistent	Chronic antigenic stimulation, immune dysregulation, and B-cell hyperactivation	[70,71]
**IgG4-related disease (IgG4-RD)**	Very limited	Co-occurrence with MGUS or related monoclonal disorders appears uncommon; evidence is limited mainly to case reports and small case series	Prevalence and prognostic significance cannot currently be reliably estimated	Chronic inflammatory and plasmacytic microenvironment may favor clonal expansion in selected cases	[75,76,77,78]
**Schnitzler syndrome**	Strong biological/clinical association	Monoclonal gammopathy, usually IgM, is an integral component of the characteristic clinical phenotype	Direct diagnostic and pathogenetic relevance; represents a paradigm of monoclonal gammopathy associated with systemic inflammation	Interaction between the monoclonal immunoglobulin/clonal B-cell process and innate inflammatory pathways	[84]

Importantly, although several clinically ascertained cohorts have reported increased frequencies of monoclonal gammopathy in individual autoimmune diseases, the population-based iStopMM screening study found no significant overall association between autoimmune disease and screen-detected MGUS after adjustment for age and sex (PR 1.05, 95% CI 0.97–1.15), suggesting that ascertainment bias may contribute to some previously reported associations [13]. Abbreviations: AIH, autoimmune hepatitis; BAFF, B-cell activating factor; HCV, hepatitis C virus; HIV, human immunodeficiency virus; IgG4-RD, IgG4-related disease; MG, monoclonal gammopathy; MGUS, monoclonal gammopathy of undetermined significance; MM, multiple myeloma; NIS, National Inpatient Sample; OR, odds ratio; PBC, primary biliary cholangitis; pSS, primary Sjögren’s syndrome; RA, rheumatoid arthritis; SLE, systemic lupus erythematosus.

**Table 2:** Selected clinical entities within the spectrum of monoclonal gammopathies of clinical significance.

Entity	Defining Feature	Typical Clonal Substrate	Principal Clinical Manifestations	Key Reference
**Monoclonal gammopathy of renal significance (MGRS)**	B-cell or plasma-cell clonal proliferative disorder producing a nephrotoxic monoclonal immunoglobulin that causes renal injury, while the underlying clone does not otherwise meet established hematological criteria for clone-directed therapy	Small plasma-cell or B-cell/lymphoplasmacytic clone; the spectrum may include MGUS-like clones, smoldering plasma-cell or lymphoplasmacytic disorders, monoclonal B-cell lymphocytosis, and selected low-grade B-cell lymphoproliferative disorders	Monoclonal immunoglobulin-associated renal lesions, including glomerular, tubular, and vascular patterns of injury; diagnosis generally requires kidney biopsy	[86]
**Monoclonal gammopathy of neurological significance (MGNS)**	Neurological disorder attributable to a monoclonal gammopathy, most commonly involving the peripheral nervous system; causality must be distinguished from coincidental coexistence of MG and neuropathy	B-cell/lymphoplasmacytic or plasma-cell clone; IgM paraproteins are particularly relevant in characteristic demyelinating neuropathies, although IgG, IgA, and light-chain gammopathies may also occur	Predominantly peripheral neuropathy; typical phenotypes include distal, symmetric, sensory-predominant demyelinating neuropathy, particularly in IgM-associated disease; anti-MAG neuropathy is a characteristic example	[87]
**Monoclonal gammopathy of rheumatologic significance (MGRhS)**	Recently proposed concept describing a non-malignant or premalignant systemic condition related to a monoclonal immunoglobulin and clonal B cells that may cause multiorgan damage or influence the clinical phenotype and management of rheumatologic disease	Usually a small B-cell, lymphoplasmacytic, or plasma-cell clone associated with a monoclonal immunoglobulin	Rheumatologic and systemic inflammatory manifestations, including phenotypes such as cryoglobulinemic vasculitis and selected autoimmune/rheumatic disorders; the monoclonal gammopathy may also affect therapeutic decision-making	[12]
**Monoclonal autoimmune gammopathies (MAGa)**	Recently proposed concept encompassing monoclonal gammopathies in which the monoclonal immunoglobulin has pathogenic autoreactivity against self-antigens and thereby contributes directly to autoimmune tissue or organ injury	Clonal B-cell or plasma-cell disorder producing a monoclonal immunoglobulin with autoantibody activity; may occur across premalignant and malignant plasma-cell/B-cell disorders	Autoimmune manifestations determined by the target antigen and affected organ system, potentially involving neural, renal, cutaneous, vascular, hematologic, or other tissues	[8]

Abbreviations: MAG, myelin-associated glycoprotein; MAGa, monoclonal autoimmune gammopathies; MG, monoclonal gammopathy; MGNS, monoclonal gammopathy of neurological significance; MGRhS, monoclonal gammopathy of rheumatologic significance; MGRS, monoclonal gammopathy of renal significance; MGUS, monoclonal gammopathy of undetermined significance.

**Table 3:** Proposed criteria for interpreting the relationship between monoclonal gammopathy and autoimmune/immune-mediated disease.

Proposed Model	Findings Supporting the Model	Findings Arguing Against/Limiting the model	Representative Clinical Setting	Strength/Limitations of Evidence
**Marker of immune activation**	MG parallels autoimmune/inflammatory activity; association with B-cell activation or disease severity	MG persists despite control of autoimmune disease; no temporal relationship	pSS and selected systemic autoimmune diseases	Predominantly observational; causality not established
**Consequence of chronic immune activation**	Autoimmune disease precedes MG; prolonged antigenic/B-cell stimulation; biologically plausible BAFF/APRIL/cytokine pathways	MG may occur independently of autoimmune activity; population-screening studies may not confirm increased prevalence	pSS, SLE, chronic HCV/HIV-associated immune stimulation	Mechanistically plausible, but direct longitudinal evidence limited
**Pathogenetic factor**	Monoclonal immunoglobulin or clone directly causes organ/tissue injury; improvement with clone-directed therapy supports causality	Mere coexistence of MG and organ manifestations is insufficient	MGRS, MGNS, MGRhS/MAGa, cryoglobulinemic manifestations	Strong in established MGCS entities; variable in emerging entities
**Coincidental association**	Older age; stable low-risk MGUS; no relationship between MG and autoimmune activity; shared background risk or ascertainment bias	Temporal/biological relationship or clone-mediated injury argues against coincidence	Incidentally detected MGUS in patients with autoimmune disease	Particularly relevant given population-based screening data

## Data Availability

No new data were created or analyzed in this study. Data sharing is not applicable to this article.

## Abbreviations

The following abbreviations are used in this manuscript:

AIH	autoimmune hepatitis
AKT	protein kinase B
ANCA	antineutrophil cytoplasmic antibody
APRIL	a proliferation-inducing ligand
AS	ankylosing spondylitis
BAFF	B-cell activating factor
BCMA	B-cell maturation antigen
Bcl-6	B-cell lymphoma 6
CHIP	Clonal hematopoiesis of indeterminate potential
CI	confidence interval
CIDP	chronic inflammatory demyelinating polyneuropathy
CXCR5	C-X-C chemokine receptor type 5
DM	dermatomyositis
HCV	hepatitis C virus
HIV	human immunodeficiency virus
HR	hazard ratio
ICOS	inducible T-cell co-stimulator
IFN-α	interferon alpha
Ig	immunoglobulin
IgA	immunoglobulin A
IgG	immunoglobulin G
IgG4-RD	immunoglobulin G4-related disease
IgM	immunoglobulin M
IL	interleukin
IL-6	interleukin 6
IL-17	interleukin 17
IL-21	interleukin 21
IL-23	interleukin 23
JAK	Janus kinase
MAGa	monoclonal autoimmune gammopathies
MAPK	mitogen-activated protein kinase
MBL	monoclonal B-cell lymphocytosis
MGCS	monoclonal gammopathy of clinical significance
MGNS	monoclonal gammopathy of neurological significance
MGRhS	monoclonal gammopathy of rheumatologic significance
MGRS	monoclonal gammopathy of renal significance
MGUS	monoclonal gammopathy of undetermined significance
MM	multiple myeloma
NAM	necrotizing autoimmune myopathy
NCI	National Cancer Institute
NF-κB	nuclear factor kappa B
NIS	National Inpatient Sample
OR	odds ratio
PANoptosis	pyroptosis, apoptosis, and necroptosis
PBC	primary biliary cholangitis
PD-1	programmed cell death protein 1
PI3K	phosphoinositide 3-kinase
PMR	polymyalgia rheumatica
POEMS	polyneuropathy, organomegaly, endocrinopathy, myeloma protein, and skin changes
RA	rheumatoid arthritis
SLE	systemic lupus erythematosus
SMM	smoldering multiple myeloma
SS	Sjögren’s syndrome
SSc	systemic sclerosis
STAT3	signal transducer and activator of transcription 3
TACI	transmembrane activator and CAML interactor
TAFRO	thrombocytopenia, anasarca, fever, reticulin fibrosis and organomegaly
T-CUS	T-cell clone of uncertain significance
Tfh	T follicular helper
Tfr	T follicular regulatory
TNF	tumor necrosis factor
TNF-α	tumor necrosis factor alpha
UCTD	undifferentiated connective tissue disease
